# Molecular Heterogeneity of the Brain Endothelium

**DOI:** 10.3390/cimb45040227

**Published:** 2023-04-16

**Authors:** Nada Alnaqbi, Mohammad G. Mohammad, Rifat Hamoudi, Aloïse Mabondzo, Rania Harati

**Affiliations:** 1 Department of Pharmacy Practice and Pharmacotherapeutics, College of Pharmacy, University of Sharjah, Sharjah P.O. Box 27272, United Arab Emirates; 2 Research Institute for Medical and Health Sciences, University of Sharjah, Sharjah P.O. Box 27272, United Arab Emirates; mmohd@sharjah.ac.ae; 3 Department of Medical Laboratories, College of Health Sciences, University of Sharjah, Sharjah P.O. Box 27272, United Arab Emirates; 4 Clinical Sciences Department, College of Medicine, University of Sharjah, Sharjah P.O. Box 27272, United Arab Emirates; rhamoudi@sharjah.ac.ae; 5 Division of Surgery and Interventional Science, University College London, London W1W 7EJ, UK; 6 Department of Medicines and Healthcare Technologies, Paris-Saclay University, The French Alternative Energies and Atomic Energy Commission, 91191 Gif-sur-Yvette, France; aloise.mabondzo@cea.fr

**Keywords:** brain endothelium, blood—brain barrier, efflux transporters, intercellular junctions, cortex, hippocampus, P-gp, bcrp, mrp-1, GLUT-1, lrp-1, Trf, claudin-5

## Abstract

The blood–brain barrier (BBB) is part of a neurovascular structure located in the brain’s micro vessels, that is essential to maintain brain homeostasis, but prevents the brain uptake of most drugs. Because of its importance in neuro-pharmacotherapy, the BBB has been the subject of extensive research since its discovery over 100 years ago. Major advances in understanding the structure and function of the barrier have been made. Drugs are re-designed to cross the BBB. However, despite these efforts, overcoming the BBB efficiently to treat brain diseases safely remains challenging. The majority of BBB research studies focus on the BBB as a homogenous structure throughout the different brain regions. However, this simplification may lead to an inadequate understanding of the BBB function with significant therapeutic consequences. From this perspective, we analyzed the gene and protein expression profiles of the BBB in the micro vessels from the brains of mice that were isolated from two different brain regions, namely the cortex and the hippocampus. The expression profile of the inter-endothelial junctional protein (claudin-5), three ABC transporters (P-glycoprotein, Bcrp and Mrp-1), and three BBB receptors (lrp-1, TRF and GLUT-1) were analyzed. Our gene and protein analysis showed that the brain endothelium in the hippocampus exhibits different expression profiles compared to the brain cortex. Specifically, brain endothelial cells (BECs) of the hippocampus express higher gene levels of abcb1, abcg2, lrp1, and slc2a1 compared to the BECs of the cortex regions with a trend of increase for claudin-5, while BECs of the cortex express higher gene levels of abcc1 and trf compared to the hippocampus. At the protein levels, the P-gp expression was found to be significantly higher in the hippocampus compared to the cortex, while TRF was found to be up-regulated in the cortex. These data suggest that the structure and function of the BBB are not homogeneous, and imply that drugs are not delivered similarly among the different brain regions. Appreciation of the BBB heterogeneity by future research programs is thus critical for efficient drug delivery and the treatment of brain diseases.

## 1. Introduction

The optimal functioning of the brain requires a stable internal environment with a controlled supply of ions and nutritive elements, such as glucose, amino acids, and vitamins. Additionally, the tight protection of the brain from potentially neurotoxic endogenous and exogenous xenobiotics is equally essential for normal brain functioning. The brain is tightly protected from the peripheral circulation by physiological barriers crucial for providing the appropriate stable environment and the strict protection required for optimal neural function. The largest barrier in the brain is the blood—brain barrier (BBB), found at the level of cerebral endothelium. This BBB maintains the brain homeostasis and the entry of xenobiotics into the brain. Dysregulation of the BBB is a key event in the pathogenesis of brain diseases, such as stroke, edema, brain traumas, and multiple sclerosis. Moreover, the BBB greatly limits drug delivery into the brain hindering the effective treatment of brain diseases [1,2,3].

The BBB is part of a neurovascular unit composed of brain endothelial cells (BECs), astrocytes, pericytes, and neurons. All of these elements contribute to the barrier function, but the key element are the endothelial cells. Because of their unique molecular, physical, transportation, and metabolic characteristics, the BECs strictly regulate the movement of molecules, ions, and cells between the blood and the brain [1,2,4]. For instance, the BECs are tightly attached together by tight inter-cellular junctional complexes that restrict the paracellular movement of molecules and ions. In addition to the tight junctions, the BECs express numerous influx/efflux transport and carrier systems on the luminal and abluminal sides. Clinically relevant efflux transporters at the BBB include members of the ATP transporter family, such as Mdr1, BCRP, and MRPs. These efflux transporters not only function as ATP-powered efflux pumps for neurotoxic substances and xenobiotics, but also for therapeutic drugs that limit drug delivery to the brain [1,2,3]. Influx transporters expressed at the BECs include the nutrient transporters that facilitate the entry of nutritive molecules into the brain. Examples include members of the solute carrier family of facilitated transporters, such as the glucose transporter slc2a1 (GLUT-1). In addition to the influx/efflux transporters, the BECs of the BBB express a number of receptor-mediated transportation systems. Some of these receptors transport molecules from the blood to the brain, such as the transferrin receptor that transports iron from the blood into the brain, while other receptors help in removing waste products from the brain. One example is the low-density lipoprotein receptor-related protein 1 (LRP1) expressed at the BECs. It plays a critical role in the brain clearance of the amyloid-beta (Aβ) peptides and its deregulation is suspected to be associated with the pathogenesis of Alzheimer’s disease [5,6]. Interestingly, due to their high expression at the BECs, many of these transport systems are being targeted to enhance drug delivery into the brain, such as the transferrin receptor (trf).

Thus, the unique molecular composition of the BECs forming the BBB allows them to protect the CNS and to regulate its homeostasis needed for proper neuronal function. However, as the BECs restrict the entry of a number of substances in the brain, they also limit the entry of therapeutic molecules that can lead to treatment failure. Because of their importance in neuro-pharmacotherapy, the BECs have been the subject of extensive research. Major advances in understanding the structure and function of the barrier have been made. Several delivery methods were developed to overcome the BBB and deliver therapeutics to the brain [7]. However, despite these efforts, overcoming the BBB efficiently to treat brain diseases safely remains challenging. A better understanding of the barrier properties in health and disease and a complete characterization of the BBB molecular components is essential to understand the mechanisms leading to its deregulation during disease and to develop better therapeutics that restore the barrier function or that cross the BBB for brain drug delivery.

For years, the blood—brain barrier has been regarded as a homogeneous structure throughout the different brain regions. However, this simplification may lead to an inadequate understanding of the BBB function with significant therapeutic consequences. A number of recent reports showed that the cellular composition of the NVU varies among the different brain regions and along the vascular tree. Therefore, differential stimuli from the immediate vascular environment may affect the BBB phenotype [8]. Indeed, endothelial heterogeneity, at the level of cell morphology and molecular composition has been described in peripheral BECs between different organs, but also within segments of the vascular loop of a same organ [9,10,11,12,13]. For instance, caveolae, which are small pits in the plasma membrane, were shown to have a higher density in the capillary endothelium compared to the arteries, arterioles, veins, or venules [14,15,16]. In the brain, heterogeneity has been described along the artery–capillary–vein axis. single-cell RNA sequencing of BECs from the brains of an adult mouse and human revealed a greater transcriptional heterogeneity in the capillary BECs compared to the arterial and venous BECs [17,18,19,20,21,22,23]. For example, BECs in capillaries express higher levels of monocarboxylate transporter 1 and Ca^2+^ ATPase Type 2 compared to the venules [24]. In addition, heterogeneity in barrier properties is known among the different brain regions specifically at the level of the circumventricular organs (CVOs). Blood vessels in CVOs, unlike the rest of the brain, are known to have fenestrated capillaries and lack the barrier property that allow the CVO to transduce information between the blood circulation and brain [25,26,27]. Furthermore, phenotypic and molecular differences have been described between astrocytes residing in gray matter (GM) compared to astrocytes in white matter (WM) in the brain. For instance, the GLUT-1 45 kDa is mainly expressed by astrocytes in the GM [28], while astrocytic end feet in GM express lower levels of GFAP compared to WM [29]. These molecular differences between astrocytes may potentially affect the brain vasculature and properties of the BBB in these areas [30]. In addition to the differences at the level of astrocytes, molecular differences have been reported in the BECs of WM compared to GM. Indeed, differences exist in the expression of the junctional proteins and these may cause differences in the BBB permeability. For example, a higher expression of occludin, claudin-5, and adherens junction α-catenin have been reported in WM compared to GM. Primary BEC cultures derived from GM are more permeable than those from WM [31]. Moreover, there are differences in the cytoskeletal structures between the vasculature in WM compared to the GM [29]. In summary, a significant heterogeneity exists in the cerebral vasculature and in the composition of NVU components between the GM and WM, and these differences could induce molecular heterogeneity in the BECs [27,32].

However, limited information exists regarding the molecular heterogeneity of BEC and the BBB physiology among the different brain regions. From this perspective, we conducted this study to analyze the gene and protein expression profile of the brain micro vessels isolated from two different brain regions, namely: the cortex and the hippocampus. We analyzed the gene and protein expression profile of inter-endothelial junctional protein (claudin-5), the ABC transporters (P-glycoprotein, Bcrp and Mrp-1), and the BBB receptors (lrp-1, TRF and GLUT-1). 

## 2. Materials and Methods

### 2.1. Animals

Adult male mice ([C57BL/6J], 8–10 weeks of age) were used in this study. The animals were obtained from the Jackson Laboratory (USA) and housed in the animal facility in the University of Sharjah (UOS). Animal handling and experiments were approved by the UOS animal care and use ethics committee. Male mice were used to overcome the gender differences. Indeed, scRNA-seq data obtained from male and female mouse BECs revealed differences in the BEC transcriptomes based on the gender, indicating that sex influences the transcription and gene expression in BECs [11,27,33].

### 2.2. Brain Capillary Isolation

Brain capillaries from the cortex and hippocampus were isolated, as previously described [34,35,36], using a combination of mechanical and enzymatic digestion and a density-gradient centrifugation. Five mice were used for each brain capillary isolation. Briefly, the brains were stored in Hanks’ balanced salt solution (HBSS) after extraction, then, they were cut in the sagittal direction into two halves. The white matter meninges and the associated vessels were removed. The tissue was then minced and centrifuged. The micro vessels were then chemically digested in 1 mg/mL collagenase dispase, 10 U/μL DNase-I, and 1 μg/mL TLCK for 1 h at 37 °C. The tissue was then pellet resuspended in HBSS containing 20% BSA, and then centrifuged at 2800 rpm for 30 min. The white matter was then discarded and the micro vessel pellets were stored at −80 °C for further analysis.

### 2.3. Purity Control of the Collected Brain Capillary

Markers of each of the two brain regions (cortex and hippocampus), selected from the “Human Brain Atlas” (protein atlas.org) were measured through real-time PCR on the whole structures. The list of primers for the brain region markers is represented in Table 1. The brain endothelial capillaries were isolated and their purity was checked by measuring the expression of the marker genes for brain endothelial cells (CD31 or PECAM), for glial cells (glial fibrillary acid protein or GFAP), and for pericytes (α-actin). The following mouse primers from Macrogen (Republic of Korea) were used: Pecam1 Mouse qPCR Primer Pair (NM_008816), F: CCAAAGCCAGTAGCATCATGGTC, R: GGATGGTGAAGTTGGCTACAGG; Gfap Mouse qPCR Primer Pair (NM_010277), F: CACCTACAGGAAATTGCTGGAGG, R: CCACGATGTTCCTCTTGAGGTG; Acta2 Mouse qPCR Primer Pair (NM_007392), F: TGCTGACAGAGGCACCACTGAA, R: CAGTTGTACGTCCAGAGGCATAG.

### 2.4. mRNA Expression Profiling of the BBB Components by Real-Time PCR

RNA Extraction. The RNeasy mini kit (Qiagen, Hilden, Germany) and Qiashredder homogenizer columns (Qiagen, Valencia, CA, USA) were used to extract RNA from the brain micro vessels. The quantity and quality of the total RNA were examined using the NanoDrop™ 2000/2000c spectrophotometer (Thermo Scientific, Lenexa, KS, USA). The A260/280 ratio ranged between 1.8 and 2.

Reverse Transcription. cDNA were synthesized using the RT2 First Strand Kit (Cat#, 330401, Qiagen, Hilden, Germany).

Quantitative Real-Time PCR. SYBR Green quantitative real-time PCR using the “Applied Biosystems^®^ StepOne™ Real-Time PCR System” and the 5x HOT FIREPol^®^ EvaGreen^®^ qPCR Supermix (Solis BioDyne, Tartu, Estonia) were used to measure the gene expression of the brain region markers, tight junctions, ABC transporters, and BBB receptors. The primers used are listed in Table 2. The PCR cycling conditions were the following: 95 °C for 12 min followed by 40 cycles of two steps (95 °C for 15 s, 60 °C for 30 s, 72 °C for 30 s). Each assay was performed in duplicate. The amplification specificity was assessed by analysis of the melting curves. The 2^−ΔCT^ method was used to calculate the relative gene expression with glyceraldehyde phosphodehydrogenase or GAPDH as the housekeeping gene [37].

### 2.5. Protein Expression Profiling of the BBB Components by Western Blot Analysis

A Western blot analysis was performed, as previously described [34,38,39,40], to determine the protein expression levels of inter-endothelial junctional protein (claudin-5), ABC transporters (P-gp, Bcrp, and Mrp-1), and BBB receptors (Lrp-1, TFR, and GLUT-1). Lysis of the cells was performed on ice with RIPA lysis buffer 1X containing a protease inhibitor cocktail followed by a centrifugation at 14,000 g for 20 min at 4 °C. Quantification of the total protein concentrations was performed using the Thermo Scientific Pierce BCA protein assay kit. Twenty μg of protein from each sample were then diluted in 1X Laemelli’s buffer solution, at 95 °C for 5 min. The total proteins were separated by electrophoresis on 12% SDS-polyacrylamide gels, transferred onto nitrocellulose membranes (Amersham, Germany) (Bio-Rad, Hercules, CA, USA), and then blocked with 7.5% skim milk in Tween tris-buffered saline (TTBS) for 1 h at room temperature. The membranes were then incubated with primary antibodies at 4 °C overnight and with secondary anti-rabbit IgG (1/3000) or anti-mouse IgG (1/3000) antibodies conjugated to horseradish peroxidase for 1 h at room temperature. The following primary antibodies from Abcam and Santa Cruz Biotechnology were used: anti-claudin-5 (ab15106); anti-Mdr-1 (sc-55510); anti-ABCG2 (sc-69989); anti-MRP1 (ab32574); anti-LRP1 (ab92544); anti-transferrin receptor (ab84036); anti-glucose transporter GLUT1 (ab652); and anti-GAPDH (ab9485) as loading and transfer controls. Visualization of the blot was performed with an enhanced chemiluminescence (ECL) kit (using the ECL™ Prime Western Blotting System GE Healthcare, cat# RPN2232 or the Bio-Rad Clarity Western ECL Substrate, cat# 1705061). The Western blots were then imaged and quantified using ChemiDoc™ Touch Imaging System (Bio-Rad).

### 2.6. Statistical Analysis

The results are expressed as the means ± SEM from three independent lots of brain endothelial micro vessels (each lot representing a pool from five mice). P values were calculated using paired Student’s t tests. The statistical significance levels were set as follows: *p* < 0.05 (*), *p* < 0.01 (**), and *p* < 0.001 (***). Calculations and figures were generated using GraphPad-Prism 8.2.0.

## 3. Results

**Figure 1:**
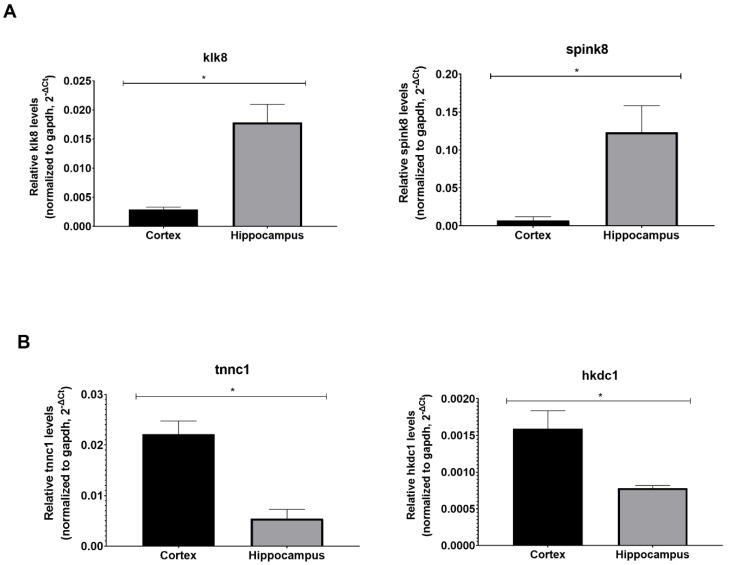
Markers of the two brain regions: the cortex and the hippocampus. mRNA levels of the brain region markers were measured in the dissected cortex and hippocampus through real-time PCR. Results are the means ± SEM from three lots of cerebral structures from three mice and the PCR performed in duplicate for each single preparation. Statistical comparisons: * *p* < 0.05.

To assess the BBB heterogeneity, we isolated brain micro vessels from two mouse brain regions: the cortex and the hippocampus. Following the brain dissection, the total RNAs were extracted from each brain region and the regions markers were measured through real-time PCR. These markers were selected from the “Human Brain Atlas” (protein atlas.org). Specifically, two hippocampal markers (klk8 and spink8) that are expressed in the hippocampus but not in the cortex, and two cortical markers (tnnc1, hkdc1) that are expressed in the cortex but not in the hippocampus were assessed through real-time PCR. The gene expression analysis of these markers validated the purity in each of the brain regions dissected, where the two markers of the hippocampus (klk8 and spink8) (Figure 1A) and those of the cortex (tnnc1, hkdc1) (Figure 1B) were, as expected, detected in their specific brain regions.

**Figure 2:**
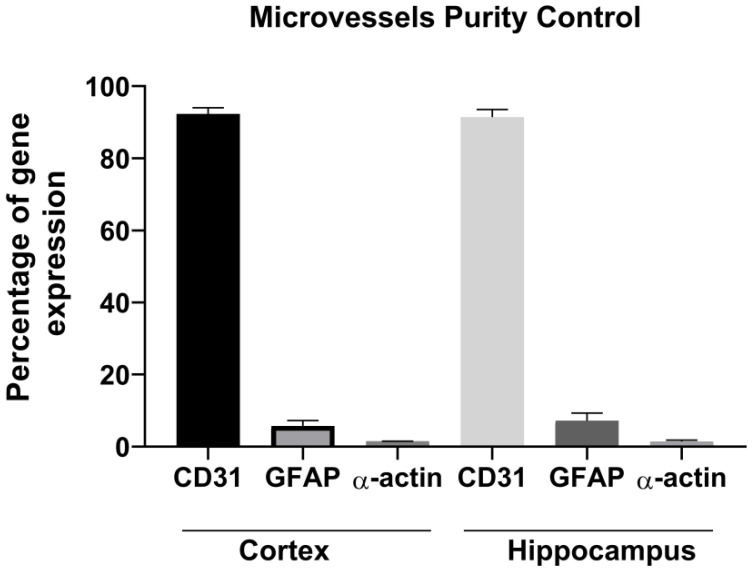
The purity of the mice brain endothelial micro vessels isolated from the cortex and the hippocampus. mRNA levels of CD3, Glial fibrillary acid, and α-actin were measured through real-time PCR. Results are the means ± SEM from three lots of brain endothelial micro vessels (each lot represents a pool from five mice) and PCR performed in duplicate for each single preparation.

We then isolated the brain capillaries from each brain region. To verify the purity of the brain capillaries, we examined the expression of the marker genes for the brain endothelial cells (CD31 or PECAM), for the glial cells (glial fibrillary acid protein or GFAP), and for the pericytes (α-actin), as previously described [35,36]. As shown in Figure 2, the isolated brain capillaries consisted mainly of brain endothelial cells; therefore, confirming the purity of the micro vessels.

**Figure 3:**
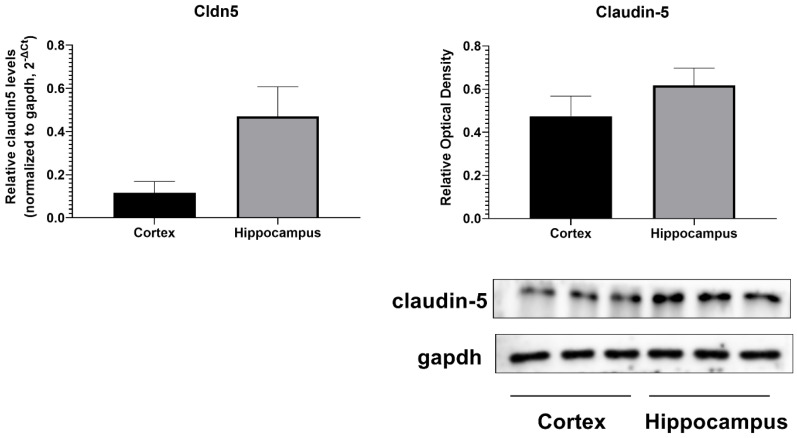
Levels of the inter-endothelial junctional protein (claudin-5) in mouse brain micro vessels isolated from the cortex and the hippocampus.

**Figure 4:**
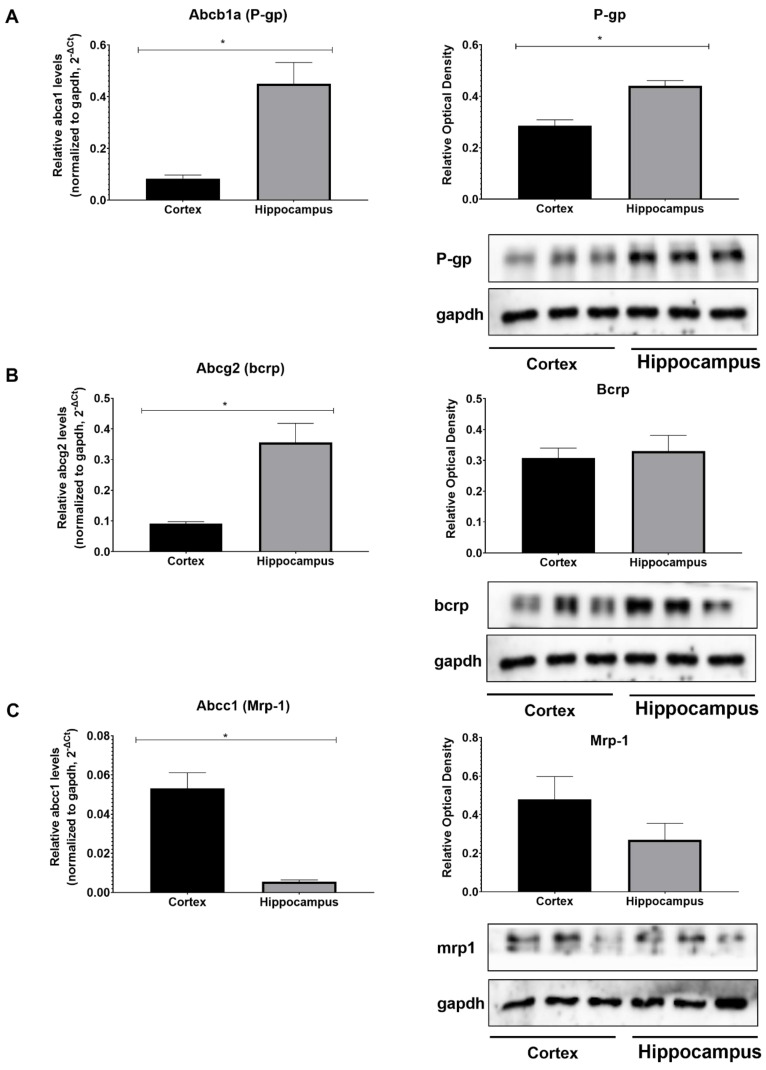
Levels of the ABC transporter proteins (P-gp, Bcrp, and Mrp1) in mouse brain micro vessels isolated from the cortex and the hippocampus. (**A**–**C**) The relative mRNA levels of abcb1a (**A**), abcg2 (**B**), and abcc1 (**C**) were assessed through real-time PCR. GAPDH was used as an internal standard. Data are represented as (2^−ΔCt^). The protein expressions of P-gp (**A**), Bcrp (**B**), and Mrp-1 (**C**) were examined using Western blot analysis. Optical densities were analyzed with Image Lab 6.0.1 software (Bio-Rad) and normalized to GAPDH. Results are the means ± SEM from three lots of brain endothelial micro vessels (each lot represents a pool from five mice). Statistical comparisons: * *p* < 0.05.

**Figure 5:**
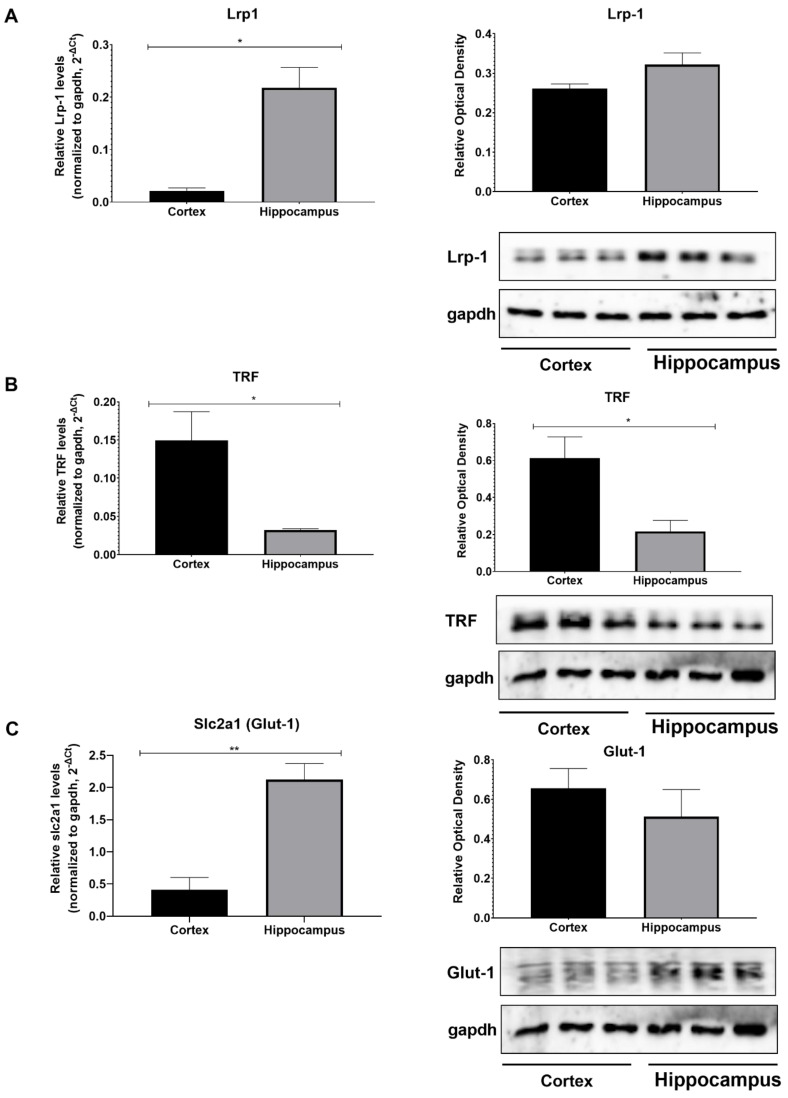
Levels of the receptor (Lrp-1, transferrin receptor and GLUT-1) in mouse brain micro vessels isolated from the cortex and the hippocampus. (**A**–**C**) The relative mRNA levels of Lrp1 (**A**), TRF (**B**), and slc2a1 (**C**) were assessed through real-time PCR. GAPDH was used as an internal standard. Data are represented as (2^−ΔCt^). The protein expressions of Lrp-1 (**A**), TRF (**B**), and GLUT-1 (**C**) were examined by Western blot. Optical densities were analyzed with Image Lab 6.0.1 software (Bio-Rad) and normalized to GAPDH. Results are the means ± SEM from three lots of brain endothelial micro vessels (each lot represents a pool from five mice). Statistical comparisons: * *p* < 0.05 and ** *p* < 0.01.

Following the verification of the purity, we measured the gene and protein expression of a number of BBB key components through real-time PCR and Western blot, respectively. Specifically, the expression profile of the inter-endothelial junctional protein (claudin-5) (Figure 3), three ABC transporters (P-glycoprotein, Bcrp, and Mrp-1) (Figure 4), and three BBB receptors (lrp-1, TRF, and GLUT-1) (Figure 5) were analyzed.

Claudin-5 is a tight junctional protein that enters in the composition of inter-endothelial tight junctions [2]. Our result showed a tendency toward the increase of the claudin-5 gene expression in the hippocampus compared to the cortex, with no significant changes observed at the protein level (Figure 3). 

The relative mRNA levels of claudin-5 were assessed through real-time PCR. GAPDH was used as an internal standard. Data are represented as (2^−ΔCt^). The protein expression of claudin-5 was examined by Western blot. Optical densities were analyzed with Image Lab 6.0.1 software (Bio-Rad) and normalized to GAPDH. Results are the means ± SEM from three lots of brain endothelial micro vessels (each lot represents a pool from five mice).

Regarding the expression of the ABC efflux transporters, our results showed a significant increase in the P-gp gene (*p* = 0.0221) and protein expression (*p* = 0.0337). The Bcrp gene expression was found to be statistically higher in the hippocampus (*p* = 0.022) compared to the cortex with no significant changes at the protein levels, while the abcc1 gene expression was found to be higher in the cortex (*p* = 0.0143) with no significant changes observed in the protein expression. These data indicate potential differential efflux activity in the two brain regions, however, this expression analysis should be complemented by efflux activity studies to confirm the differential efflux activity.

Expression analysis of the BBB receptors Lrp1, trf, and GLUT-1 showed that the gene expressions of lrp1 and GLUT-1 are significantly higher in the hippocampus compared to the cortex (*p* = 0.0182; *p* = 0.0092, respectively), while the trf gene and protein expression were found to be significantly lower in the hippocampus compared to the cortex (*p* = 0.042; *p* = 0.0334, respectively) indicating a heterogeneity of the BBB receptors’ expression in different brain regions. Similarly, this expression analysis merits further activity analysis.

## 4. Discussion

A very interesting and important appearance is the up-regulation of a gene expression in one region and its down-regulation in the other brain region. Brain endothelial cells (BECs) of the hippocampus express higher gene levels of abcb1, abcg2, lrp1, and slc2a1 compared to the BECs of the cortex regions, with a trend of increase for claudin-5, while the BECs of the cortex express higher gene levels of abcc1 and trf compared to the hippocampus. At the protein levels, P-gp expression was found to be significantly higher in the hippocampus compared to the cortex, while TRF was found to be up-regulated in the cortex. Intriguingly, these experiments demonstrated a molecular heterogeneity of the brain endothelium and revealed that the hippocampus may have a modified blood—brain barrier compared to the cortex, which indicates that the different brain regions may have different accessibility to pharmacological agents due to the BBB regional heterogeneity.

Our results showed a tendency towards an increase in claudin-5 expression in the hippocampus. Claudin-5 is a key, mostly enriched tight junctional protein that controls the paracellular permeability of the BBB and is vital for maintaining the brain endothelium integrity [41,42]. Dysregulations of claudin-5 as well as other junctional proteins are known to be a key event in the pathogenesis of a range of brain diseases, including inflammatory diseases, such as multiple sclerosis (MS) [42]. Indeed, in MS, dynamic tight junction remodeling and alteration in claudin-5 expression cause the breakdown of the brain endothelial barrier, that allows for the transendothelial migration of leukocytes into the brain and progression of the disease. Intriguingly, differences in the BBB disruption between the WM and GM during MS have been reported, where disruption of the barrier and infiltration of immune cells is most notable in the WM vasculature compared to the GM [43,44,45,46,47]. These differences in MS representation is most likely due to the vasculature heterogeneity in the WM compared to the GM. 

In HIV-1 infected patients, the virus enters the CNS leading to neurocognitive impairments. A down-regulation of the TJ complex components, including claudin-5, was shown in vitro and in vivo, which leads to enhanced monocytes infiltration into the brain contributing to the neurocognitive impairments [48,49]. Interestingly, a brain autopsy revealed that the brain vasculature in WM is more compromised than in the GM, where expressions of the junctional proteins occludin and ZO-1 were more disrupted or even absent in the WM [50]. Heterogeneity in the brain vasculature could be involved in the manifestation of the differential pathologies in various brain regions [32].

In rodent models of stroke and traumatic brain injury, claudin-5, occludin, and ZO-1 were down-regulated with the opening of the BBB leading to extravasation of serum proteins [51]. A better understanding of the expression and functional profile of junctional proteins at the BBB among the different brain regions will provide new insights into the understanding of the pathogenesis of incurable brain diseases characterized by increased permeability of the barrier, allowing for the development of optimized therapeutic strategies. Furthermore, therapeutic targeting of claudin-5 has been under investigation, where claudin-5 could be transiently down-regulated for a temporary increase in BBB permeability. For instance, RNA interference has been tested in models of traumatic brain injury and Alzheimer’s disease to protect the brain against entry of neurotoxic material or to enhance the delivery of small therapeutic molecules to the brain [52,53,54,55]. As these approaches are of considerable interest to increase the size-exclusion limits of the brain endothelium by transiently down-regulating claudin-5, an appreciation of the differential expression of claudin-5 among the different regions of the brain endothelium is needed for better outcomes of these therapeutic approaches, aiming to selectively down-regulate claudin-5.

The ABC transporters, including abcb1, abcg2, and abcc1 are known to be enriched at the BBB and they play a key role in maintaining the brain homeostasis by actively hindering the entry into the CNS of potentially neurotoxic xenobiotics circulating in the blood [56]. Moreover, the high expression of these efflux transporters at the BBB are considered a major impediment in the treatment of brain diseases as they represent an obstacle in delivering therapeutic drugs to the brain which limits their effectiveness [57,58,59,60]. Our results show a differential expression of these between the cortex and the hippocampus, implying that some brain regions may be more accessible to therapeutic drugs compared to others. These results merit further functional studies to confirm the differential efflux activity. This regional heterogeneity should be considered when designing therapeutic strategies to treat brain diseases. For instance, our results showed that P-gp expression was higher in the hippocampus compared to the cortex. P-gp is a well-studied protein due to its important role in protecting the tissues against xenobiotics. Moreover, P-gp overexpression is a major contributor to the multi-drug resistance phenomenon [61], where this glycoprotein exports numerous molecules, including chemotherapeutic drugs, such as doxorubicin or vinblastine, natural products, including flavonoids, and opioids, such as morphine and others [58,61]. Due to its important protective role and contribution in the MDR, appreciating the heterogeneity of P-gp expression among the different regions will help in developing better therapeutic strategies to deliver drugs to the brain, and will allow for a better understanding of the pathogenesis of many brain diseases. Indeed, P-gp dysregulation has been implicated in a number of neurological disorders, including Alzheimer’s disease [62,63], HIV encephalitis [64], Parkinson’s disease [65], and epilepsy [66,67,68]. Our results showed a higher gene expression of bcrp in the hippocampus compared to the cortex, while a higher expression of mrp1 was noted in the cortex. In inflammation, Mrp1 transports inflammatory mediators [leukotriene C4 (LTC4), prostaglandin E2 (PGE2)]. Similar to P-gp, MRP1 takes in charge a broad number of endogenous and exogenous metabolites and substrates, and is involved in the MDR phenomenon leading to treatment failure [58,69,70]. Bcrp, another clinically relevant ABC transporter expressed at the BBB, is involved in the efflux of cytotoxic drugs (topotecan and ofloxacin) and toxic compounds present in food [2-amino-1-methyl-6-phenylimidazo(4,5-b)pyridine (PhIP)], among others [71]. Similar to P-gp and MRPs, bcrp helps in protecting the brain against the entry of xenobiotics, but also compromises the therapeutic efficacy of CNS drugs [58,72]. Because of the important role of ABC transporters in protecting the brain against MDR, treatment failure, and in the pathogenesis of brain diseases, a better understanding of their expression at the BBB in the different brain regions will help in better understanding the pathogenesis of brain diseases and in developing optimized strategies to treat CNS disorders. 

A number of receptors are expressed at the BBB and help in mediating the receptor mediated transcytosis of substances across the barrier. These receptors include the lipid transporter LRP1, the iron transporter transferrin receptor, and the glucose transporter GLUT-1. Our results showed a higher expression of Lrp-1 in the hippocampus compared to the cortex. Lrp-1 has been shown to transport multiple ligands across the BBB, including amyloid β1-42 (Aβ) [73], and it helps in maintain the BBB integrity [73]. Dysregulation of Lrp-1 at the BBB has been shown to contribute to neurotoxic amyloid-β (Aβ) brain accumulation of Aβ in the brain, which drives the Alzheimer’s disease pathology. Clearance of Aβ mediated by lrp-1 at the BBB has been suggested as a potential target for treatment and prevention of Aβ brain accumulation in Alzheimer’s disease [73], and therefore a better understanding of the lrp-1 expression at the BBB will help in optimizing such a therapeutic strategy.

One strategy to deliver therapeutic cargos across the BBB consists of using antibodies and peptide ligands against various receptors expressed at the BBB level, and that are involved in endocytosis [74,75]. Trf is highly expressed at the brain endothelium and is regarded as a potential target for drug delivery to the brain [76]. Similarly, GLUT-1, which is abundantly present at the brain endothelium, is regarded as an effective target to deliver the brain-derived neurotrophic factor gene across the BBB [77]. Our results showed a differential expression of lrp1, trf, and GLUT-1 in the cortex compared to the hippocampus. Functional studies are needed to confirm the differential passage of substances in the different brain regions. However, these results suggest that the heterogeneity in the expression of these receptors mediating transcytosis should be considered in translational studies for antibody-based BBB delivery strategies.

As for the mechanisms and factors leading to the molecular heterogeneity of the BECs, the BEC phenotype is known to be influenced by many factors, including the surrounding brain environment and other cells of the NVU. Indeed, the cellular composition of the brain parenchyma surrounding the BEC varies between different brain regions, for example between the white and gray matter, which may lead to this molecular heterogeneity. As mentioned, the barrier property is known to be regulated by other cells of the NVU. For instance, astrocytes are essential to induce and maintain the TJ and therefore the integrity of the brain endothelial barrier [78,79,80]. Differences in astrocyte phenotypes among the different brain regions have been reported, and this could also be a factor contributing to the molecular heterogeneity observed [27]. Pericytes, another component of the BBB, have also been shown to increase the BBB integrity [81,82] and the proportion of TJ proteins (occludin and claudin-5) [83]. A deficiency of pericytes reduced the expression of the junctional proteins ZO-1, occludin, and claudin, and VE-cadherin [84], but resulted in a heterogeneous increase in the barrier’s permeability across the CNS [85], where the high increase in permeability was observed in the cortex, striatum, and hippocampus with lower changes observed in the diencephalon, midbrain, and cerebellum [86]. Another important part of the NVU is the extracellular matrix (ECM), that also influences the BEC phenotypes and functions. ECM is composed of collagens, laminins, fibrillins, fibronectin, and vitronectin that play a play an important role in regulating the BBB integrity [87,88]. Differences in ECM composition among the different brain regions may also be a contributing factor leading to the BE heterogeneity. A better understanding of the heterogeneity of the NVU components among the different brain regions will help in better understanding the factors contributing to the heterogeneity in the endothelial barrier. In addition to the NVU components influencing the barrier’s property, the differential activation of the signaling pathways that are known to regulate the barrier’s property, such as Wnt/β-catenin [89], among the different brain regions should be investigated.

## 5. Limitations of the Study

In this study, we analyzed the gene and protein expression of a number of BBB components between two brain regions, the cortex and the hippocampus. This expression analysis should be complemented with functional tests and efflux activity studies to confirm the differential efflux activity. Furthermore, single-cell RNA sequencing will characterize in more detail the transcriptional changes at the BE of the hippocampus compared to the cortex. Differences among other brain regions should also be investigated. In addition, the development of a region specific in vitro BBB model that includes the different components of the NVU [32] will allow for a better understanding of the molecular mechanisms and factors contributing to the molecular heterogeneity of the brain endothelial barrier.

## 6. Conclusions

Analysis of the gene and protein expressions revealed that some structures in the normal hippocampus of animals had different quantitative patterns of receptors and transporters (efflux and influx) compared to the non-neurogenic brain regions, such as the cortex. Thus, our results indicate that the different brain regions are heterogeneous and that the expression of the receptors and transporters is not uniform throughout the brain. These results indicate a pathway for further exploration of the regional differences in cerebral substrate transportation. A comprehensive analysis of the molecular heterogeneity between the brain regions would allow for a better understanding of the pathogenesis of brain diseases. A further appreciation of such heterogeneity will be critical for the efficient therapeutic targeting of the endothelium and the management of central nervous system diseases.

## Figures and Tables

**Table 1:** List of primers used for the transcriptional profiling of the brain region markers through real-time PCR.

Klk8 Mouse qPCR Primer Pair (NM_008940): Hippocampus marker	
Forward Sequence	TCCTGGTTGGAGACAGATGGGT
Reverse Sequence	AGGATGCTGGATAGACTGAGCC
Spink8 Mouse qPCR Primer Pair (NM_183136): Hippocampus marker	
Forward Sequence	CATCCTTCCTATGAACTTTCACATG
Reverse Sequence	CTCGTAGGTCACTTGGTTGCTG
Tnnc1 Mouse qPCR Primer Pair (NM_009393): Cortex marker	
Forward Sequence	GATGGTTCGGTGCATGAAGGAC
Reverse Sequence	CTTCCGTAATGGTCTCACCTGTG
Hkdc1 Mouse qPCR Primer Pair (NM_145419): Cortex marker	
Forward Sequence	TTTCGCTCAGCCAATCTCTGCG
Reverse Sequence	ACCACCTTGTGTAGGCGTTTGG

**Table 2:** List of primers used for the transcriptional profiling of the BBB components through real-time PCR.

Gapdh Mouse qPCR Primer Pair (NM_008084)	
Forward Sequence	CATCACTGCCACCCAGAAGACTG
Reverse Sequence	ATGCCAGTGAGCTTCCCGTTCAG
Cldn5 Mouse qPCR Primer Pair (NM_013805)	
Forward Sequence	TGACTGCCTTCCTGGACCACAA
Reverse Sequence	CATACACCTTGCACTGCATGTGC
Abcb1a Mouse qPCR Primer Pair (NM_011076)	
Forward Sequence	TCCTCACCAAGCGACTCCGATA
Reverse Sequence	ACTTGAGCAGCATCGTTGGCGA
Abcg2 Mouse qPCR Primer Pair (NM_011920)	
Forward Sequence	CAGTTCTCAGCAGCTCTTCGAC
Reverse Sequence	TCCTCCAGAGATGCCACGGATA
Abcc1 Mouse qPCR Primer Pair (NM_008576)	
Forward Sequence	CAGTGGTTCAGGGAAGGAGTCA
Reverse Sequence	CACTGTGGGAAGACGAGTTGCT
Lrp1 Mouse qPCR Primer Pair (NM_008512)	
Forward Sequence	CGAGAGCCTTTGTGCTGGATGA
Reverse Sequence	CGGATGTCCTTCTCAATGAGGG
Tfrc Mouse qPCR Primer Pair (NM_011638)	
Forward Sequence	GAAGTCCAGTGTGGGAACAGGT
Reverse Sequence	CAACCACTCAGTGGCACCAACA
Slc2a1 Mouse qPCR Primer Pair (NM_011400)	
Forward Sequence	GCTTCTCCAACTGGACCTCAAAC
Reverse Sequence	ACGAGGAGCACCGTGAAGATGA

## Data Availability

Data Availability Statements are available in the manuscript.
